# Utilization of Enzyme-Immobilized Mesoporous Silica Nanocontainers (IBN-4) in Prodrug-Activated Cancer Theranostics

**DOI:** 10.3390/nano5042169

**Published:** 2015-12-04

**Authors:** Bau-Yen Hung, Yaswanth Kuthati, Ranjith Kumar Kankala, Shravankumar Kankala, Jin-Pei Deng, Chen-Lun Liu, Chia-Hung Lee

**Affiliations:** 1 Department of Life Science and Institute of Biotechnology, National Dong Hwa University, Hualien-974, Taiwan; E-Mails: baofungi@gmail.com (B.-Y.H.); yaswanthk1987@gmail.com (Y.K.); ranjithkankala@yahoo.com (R.K.K.); cliu@mail.ndhu.edu.tw (C.-L.L.); 2 Department of Chemistry, Kakatiya University, Telangana State-506009, India; E-Mail: shravankankala@yahoo.com; 3 Department of Chemistry, Tamkang University, New Taipei City 251, Taiwan; E-Mail: jpdeng@mail.tku.edu.tw

**Keywords:** mesoporous silica nanoparticles, enzyme immobilization, Institute of Bioengineering and Nanotechnology (IBN-4), anti-cancer, prodrug therapy

## Abstract

To develop a carrier for use in enzyme prodrug therapy, Horseradish peroxidase (HRP) was immobilized onto mesoporous silica nanoparticles (IBN-4: Institute of Bioengineering and Nanotechnology), where the nanoparticle surfaces were functionalized with 3-aminopropyltrimethoxysilane and further conjugated with glutaraldehyde. Consequently, the enzymes could be stabilized in nanochannels through the formation of covalent imine bonds. This strategy was used to protect HRP from immune exclusion, degradation and denaturation under biological conditions. Furthermore, immobilization of HRP in the nanochannels of IBN-4 nanomaterials exhibited good functional stability upon repetitive use and long-term storage (60 days) at 4 °C. The generation of functionalized and HRP-immobilized nanomaterials was further verified using various characterization techniques. The possibility of using HRP-encapsulated IBN-4 materials in prodrug cancer therapy was also demonstrated by evaluating their ability to convert a prodrug (indole-3-acetic acid (IAA)) into cytotoxic radicals, which triggered tumor cell apoptosis in human colon carcinoma (HT-29 cell line) cells. A lactate dehydrogenase (LDH) assay revealed that cells could be exposed to the IBN-4 nanocomposites without damaging their membranes, confirming apoptotic cell death. In summary, we demonstrated the potential of utilizing large porous mesoporous silica nanomaterials (IBN-4) as enzyme carriers for prodrug therapy.

## 1. Introduction

The delivery of fragile drugs to target sites at precise times and locations with high drug activity in a reproducible manner is an outstanding challenge. Various delivery approaches have been developed to create effective therapeutic methods to target cancerous sites using a variety of versatile drug formulations. The magic of bioactivation is that the therapeutic method acts by specifically activating the prodrug into active drug molecules upon reaching the targeting site. The prodrug approach was developed by Albert in 1958 for the purposes of increasing therapeutic efficacy and reducing cytotoxic side effects through the activation of toxic drugs at targeted sites. A prodrug is defined as a nontoxic precursor of an active drug that can be transformed into the active drug molecule by enzymatic catalysis and consequently deliver therapeutic effects to targeted sites. Enzyme prodrug therapy (EPT) is a novel therapeutic approach where prodrug-activating enzymes are initially delivered into the cancer cells using various targeting approaches; this enzyme delivery is followed by treatment with a nontoxic prodrug that is specifically activated into an anticancer drug through the enzymatic activity in the targeted cells. The concentration of the activated drug can be high in the tumor sites, thereby reducing systemic toxicity in normal tissues [1,2,3,4,5]. Hence, this therapeutic method can be applied to improve tumor targeting while reducing systemic toxicity. For the successful use of EPT in clinical applications, the catalytic enzymes in normal tissues that activate the prodrugs must be present at lower concentrations than in cancer tissues [6,7]. In recent years, EPT has been examined as a potential strategy for treating devastating diseases such as cancer [8,9,10]. The development of an efficient enzymatic delivery vehicle that can carry and activate prodrug molecules and kill cancer cells is extremely important. Many approaches have been developed to deliver various enzymes for the activation of anticancer prodrugs, such as antibodies, viruses, lectins, spores and liposomes [11,12,13,14,15,16,17,18,19]. Despite some of the advantages associated with these approaches, the sole use of antibodies and lectins is hindered by their poor bioavailability [20], whereas the scope of possible pathogenicity hampers the use of microbial vectors. However, the use of immunoliposomes has been shown to enhance enzymatic delivery compared to the sole use of antibody-enzyme conjugates [21,22], which has gained considerable attention with regards to nanoparticle-based drug delivery systems. Motivated by the advantages of immunoliposomes observed in drug delivery, many researchers have used liposomal, polymeric and other organic nanoparticle-based systems for prodrug therapy [23,24,25,26,27]. Nevertheless, liposomes and other organic nanoparticles have also been shown to have certain drawbacks, such as low encapsulation efficiencies, rapid leakage of water-soluble molecules, susceptibility to microbial attack in the presence of blood components and poor storage stabilities; these drawbacks may limit the application of liposomes in prodrug therapy [28].

In the last decade, exceptional developments have been made with respect to inorganic nanoparticles in the field of drug delivery. Researchers have developed various prodrugs for targeted delivery using a wide range of inorganic nanocomposites [17,29,30,31,32]. Many inorganic nanomaterials such as gold [33], silver, magnetic-Fe/Fe_3_O_4_ [34], gold-coated super paramagnetic iron oxide [35], carbon nanotubes, mesoporous silica [36,37], and layered double hydroxide (LDH) nanoparticles [38,39] have gained much attention due to their novel multifaceted characteristics that include low cytotoxicities, high loading capacities, large surface to volume ratios, good stabilities, rich functionalities, biocompatibilities, and targeting abilities; consequently, these types of inorganic nanomaterials are considered to be ideal enzyme carriers for various biological applications.

Many breakthroughs have been made for the loading of bioactive molecules such as enzymes placed in the tunable pores of mesoporous silica and great improvements in cellular uptake efficiencies [40,41]. Very recently, phosphonate@mesoporous silica nanoparticles (MSNs) were synthesized for the intracellular delivery of fluorescein-labeled bovine serum albumin (BSA) cargo using the folate receptor pathway; these MSNs were shown to have potential for use in targeted drug delivery for the treatment of cancer [42]. Sun *et al.* reported the development of gold nanoparticle-capped MSNs loaded with luciferase to retain enzyme bioactivity through intracellular-controlled catalysis and their use in tumor imaging. This provides a unique platform for monitoring metastasis by means of bioluminescence and using intracellular ATP and GSH levels as indicators [43]. Mou *et al.* proposed a new strategy for the intracellular delivery of superoxide dismutase, where multi-functional MSN nanocontainers were used to enhance the transmembrane permeability of the enzyme. This therapy was successful in delivering protein treatment for ROS-mediated diseases [44]. Interestingly, a variety of enzymes were immobilized in the mesoporous silica spheres in high loads, with safeguards to maintain stability [45,46,47,48], and the enzymes were shown to retain their bioactivities [41,49,50].

**Figure 1:**
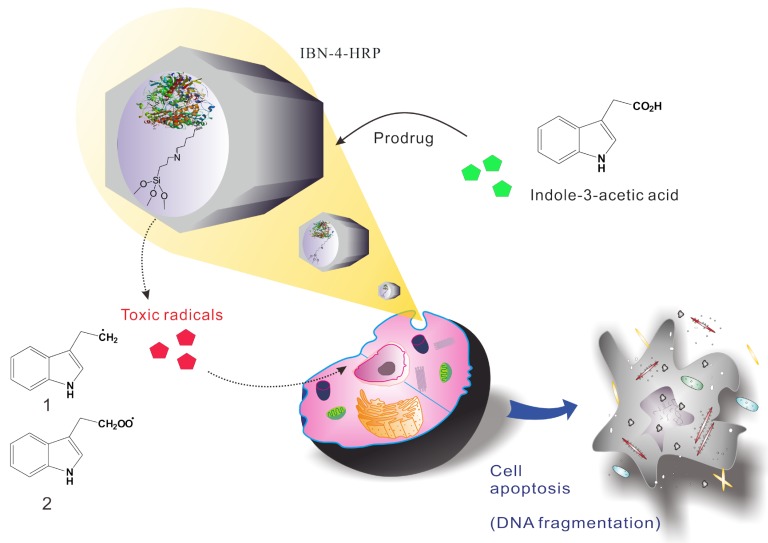
Schematic representation of enzyme prodrug therapy using IBN-4-HRP nanocomposites in the presence of indole-3-acetic acid and the resultant cell apoptosis (**1**. Skatolyl radical and **2**. Peroxyl radical).

For the successful exploitation of EPT in treating cancer, it is a crucial to maintain therapeutic levels of the enzymes for catalytic activity at the tumor sites; simultaneously, the prodrug must be able to cross the cell membranes for successful prodrug activation. To meet these criteria, there is a need to employ safe delivery vehicles to protect the enzymes from biological stress and to retain their catalytic activity. Furthermore, the carriers must be able to selectively transport the enzymes to the desired sites after crossing the cellular membranes to maintain therapeutic levels of the prodrug and the enzyme even after prolonged circulation times. These outstanding and largely unmet challenges are best addressed by MSNs (Figure 1). The immobilization of enzymes in MSNs for use in EPT is one of the most promising developments in cancer therapy. MSNs have many advantages including high biocompatibility, easy biodegradation and metabolism, controllable particle and pore sizes [51,52,53,54,55,56], large surface areas [57], and an abundance of sites available for surface functionalization, which render them the most preferable enzyme delivery vehicle to date [58]. Earlier studies have shown that immobilization of enzymes in mesoporous silica are less susceptible to damage caused by changes in pH and temperature and interactions with organic solvents [46,59,60,61,62]. Enzymes can be safely transported to target sites by encapsulation in the nanochannels of mesoporous silica; the scope of these vehicles with regards to targeting tumor cells can be expanded by attaching various types of ligands (e.g., antibodies, peptides, aptamers, and vitamins) to the surface of the MSNs [63,64].

## 2. Results and Discussion

**Figure 2:**
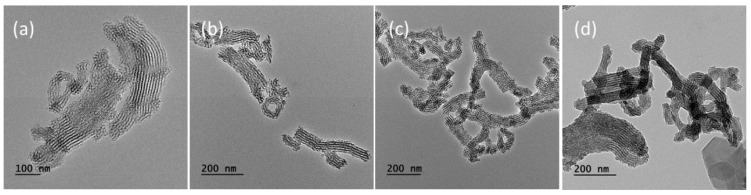
Transmission electron microscopic images of IBN-4 nanoparticles: (**a**) As-synthesized IBN-4, (**b**) IBN-4-extracted, (**c**) IBN-4-NH_2_, and (**d**) IBN-4-HRP.

A schematic illustration for the synthesis of enzyme-immobilized MSNs is shown in Figure 1. The purpose of our study was to immobilize HRP onto IBN-4 nanoparticles via covalent linkages to facilitate the activation of the prodrug IAA (indole acetic acid) in the tumor microenvironment. Pristine IBN-4 nanoparticles were synthesized using a template (Pluronic P123) and an FC-4 nanoparticle overgrowth protectant (fluorocarbon surfactant). Hydrolysis and condensation of tetraethoxysilane (TEOS) were performed under dilute acidic conditions. The surfactant was then removed by oxidation with ammonium perchlorate and a 10 M HNO_3_ solution; the resulting sample was labelled “IBN-4-extracted”. After oxidation under acidic conditions, the surfaces had large amounts of silanol groups, which were easily functionalized with 3-aminopropyltrimethoxysilane (APTS) to afford the amine-modified mesoporous silica surfaces (IBN-4-NH_2_). Subsequent conjugation of glutaraldehyde (GA) (IBN-4-NH-GA) allowed the aldehyde to serve as an anchor for the successful immobilization of enzymes on the surface of the silica particles through the formation of imine bonds (IBN-4-HRP). The reaction between IAA and HRP proceeded through a complex mechanism where IAA was activated by HRP and produced free radicals (Figure 1), such as indolyl, skatolyl, and peroxyl radicals, and reactive oxygen species (ROS), such as ·O_2_^−^ and H_2_O_2_. These reactive molecules caused morphological changes of the cells and initiated damage by inducing apoptotic-signaling cascades [65]. Thus, the proposed IAA/HRP combination may have enhanced cellular oxidative stress and may have led to cell death. Nanoparticle size and enzyme stability play a key role in the interaction and activation of prodrug molecules in cells during EPT. First, nanoparticles within a range of <300 nm were shown to have good dispersion, escape uptake by the reticuloendothelial system (RES) and enhance the EPR (Enhanced permeability and retention) effect [66,67,68]. However, recent research has shown that nanoparticles less than 100 nm in size could result in severe cytotoxicity due to nonspecific cellular interactions [69,70,71]. Therefore, in the current investigations, we employed IBN-4 nanoparticles that had a mean diameter that was greater than 100 nm, as shown in Figure 2. It was evident from the Transmission electron microscopic (TEM) images that the particles exhibited an average particle diameter of approximately 150–300 nm with aspect ratios (length to the width dimension ratio of uniformly sized nanoparticles) in the range of approximately 4 to 6. The morphology and particle size of IBN-4 did not change after the amine modification process (Figure 2c) and subsequent conjugation of HRP (Figure 2d). In addition, the DLS measurements (particle diameter and zeta potential) were recorded and are illustrated in Table 1. The particle sizes of all of the samples were nearly in accord with observations from the TEM images; however, a small amount of aggregation was observed in the case of IBN-4-HRP, which resulted in aggregates sizes of ~390 nm. Previous reports suggest that particles with an average particle size of <300 nm should be favorable due to ease of internalization considering the EPR effect [66,56]. The zeta values of pristine IBN-4 and IBN-4 extracted were −0.1 and −10.9 mV, respectively. IBN-4-HRP exhibited a negative shift in surface charge (*i.e.*, +17.7 mV) compared to IBN-4-NH_2_ (*i.e.*, +26.3 mV). The positive zeta potential of enzyme-loaded IBN-4 nanoparticles facilitated cellular internalization through negatively charged cell membranes during biological investigations.

**Table 1:** Physical properties of the mesoporous materials.

Sample	BET Surface Area (m^2^/g)	Pore Size (nm)	Pore Volume (cm^3^/g)	Particle Size * (nm)	Zeta Potential * (mV)
Pristine IBN-4	345	7.9	0.75	271 (±3.0)	−0.1 (±0.1)
IBN-4 extracted	812	9.3	1.71	254 (±7.6)	−10.9 (±0.4)
IBN-4-NH_2_	357	6.0	0.98	296 (±6.4)	+26.3 (±0.7)
IBN-4-HRP	296	6.5	1.1	394 (±9.5)	+17.7 (±0.2)

* Values in parenthesis represent the standard deviation for the respective mean value.

**Figure 3:**
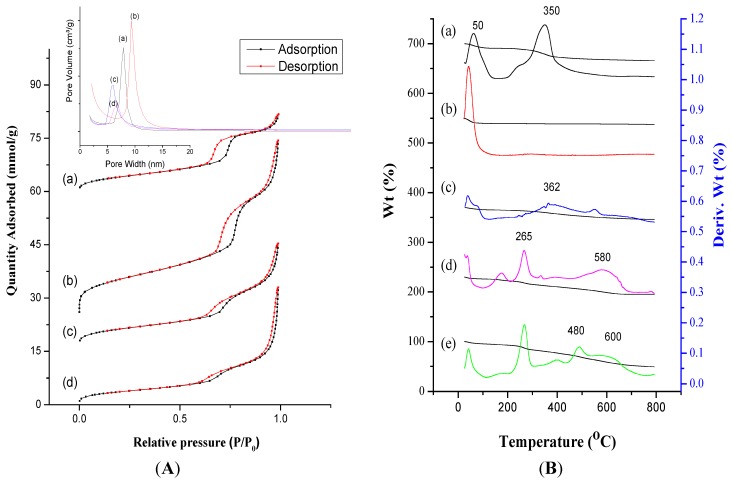
(**A**) Nitrogen adsorption–desorption isotherms of (**a**) as-synthesized IBN-4, (**b**) IBN-4 extracted, (**c**) IBN-4-NH_2_ and (**d**) IBN-4-HRP. Corresponding pore size distribution plots are shown in the inset figure. (**B**) Thermogravimetric analysis curves of (**a**) as-synthesized IBN-4; (**b**) IBN-4 extracted, (**c**) IBN-4-NH_2_, (**d**) IBN-4-NH-GA and (**e**) IBN-4-HRP.

The mesoporous characteristics of IBN-4 nanoparticles were determined using N_2_-adsorption/desorption isotherms and BET theory with an ASAP 2020 Micromeritics surface area analyzer at −196 °C (Figure 3A, Table 1). The pore size distributions of pristine IBN-4, IBN-4 nanoparticles after surfactant removal (IBN-4-extracted), IBN-4 nanoparticles modified with APTS groups (IBN-4-NH_2_) and glutaraldehyde conjugation nanoparticles for immobilization of HRP (IBN-4-HRP) were determined from adsorption branches of the isotherms using the Barrett–Joyner–Halenda (BJH) method (inset of Figure 3A). There was a significant increase in the specific surface areas and pore volumes of the nanoparticles after surfactant removal from as-synthesized IBN-4 (345 m^2^·g^−1^, 0.75 cm^3^·g^−1^) (Figure 3A(a)) to 812 m^2^·g^−1^ and 1.71 cm^3^·g^−1^, respectively (Figure 3A(b)). Later, the final BET surface areas of the IBN-4-extracted sample decreased from 812 to 357 m^2^·g^−1^ and the pore volumes decreased from 1.71 cm^3^·g^−1^ to 0.98 cm^3^·g^−1^ after amine modification (Figure 3A(c)). These changes were sufficient for the conjugation of enzymes in the mesoporous channels. Furthermore, the immobilization of the HRP enzyme (Figure 3A(d)) through a glutaraldehyde anchor decreased the surface area to 296 m^2^·g^−1^, but the pore volume was increased to 1.1 cm^3^·g^−1^ due to the contributions of enzyme molecules to the porosity of the particles. In addition, the area and intensity of the pore size distribution near 6.0 nm were further decreased, which showed that parts of the nanochannels were now occupied by enzymes.

Thermogravimetric (TGA) curves of all of the samples were recorded and the successive weight loss events were analyzed, as illustrated in Figure 3B. The primary weight loss (*i.e.*, 5%–8%) below 100 °C was attributed to loss of physisorbed moisture of the sample (Figure 3B(a)–(e)). The weight loss observed at 350 °C was due to surfactant decomposition in the as-synthesized sample (Figure 3B(a)) and the same observation was made for the IBN-4 sample extracted with ammonium perchlorate (Figure 3B(b)), which revealed the complete extraction of the surfactant. Similarly, a broad decomposition band centered at 362 °C was due to weight loss (*i.e.*, ~16%) from decomposition of the surface functionalized APTS. Furthermore, conjugation of glutaraldehyde to the surface amine resulted in decomposition at 180, 265 (*i.e.*, ~12%) and 580 °C. This confirmed the effective conjugation of the glutaraldehyde spacer for immobilization of HRP. The final events in the HRP-immobilized samples (IBN-4-HRP (Figure 3B(e)) resulted in weight loss in the range of 400–500 °C, demonstrating the effective immobilization of the enzymes (HRP weight loss alone *i.e.*, ~14%) that completely occupied the active aldehyde groups. It was detrimental for any of the aldehyde groups to remain free because glutaraldehyde exists as a low molecular weight polymer when it is used as spacer for protein immobilization. Thus, researchers should be quite cautious when using glutaraldehyde as a spacer. The amount of HRP weight loss from IBN-4 was approximately the same as the loading percentage obtained from ultraviolet-visible (UV-Vis) spectroscopy using a Bio-Rad enzyme quantification assay (IBN-4-HRP (13.13%)).

**Figure 4:**
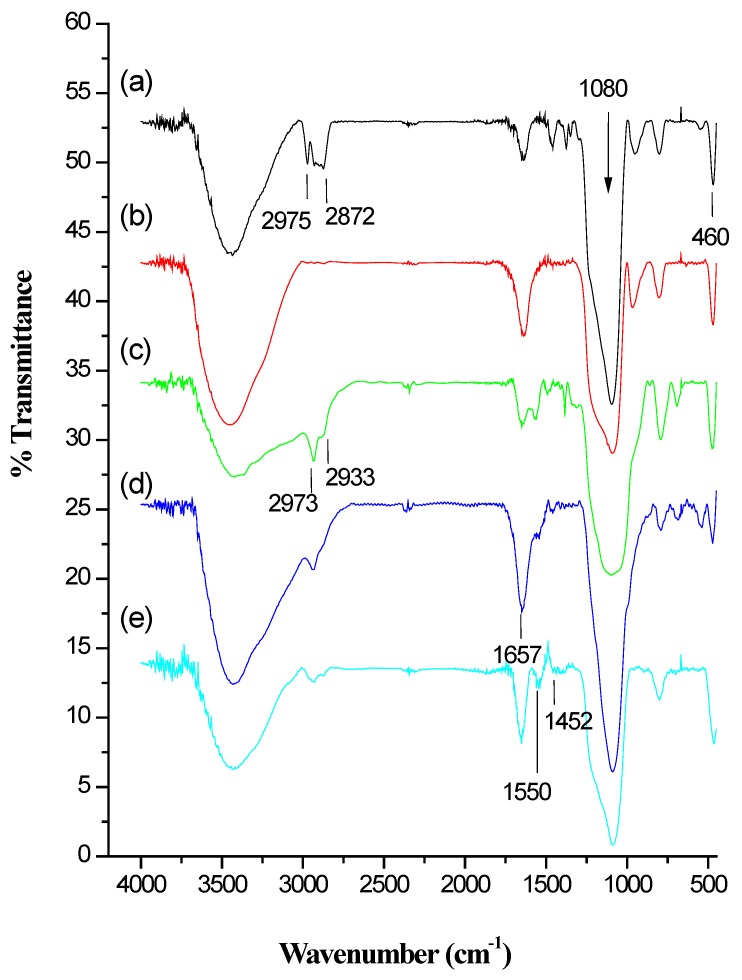
Fourier transform infrared spectroscopy (FT-IR) spectra of the (**a**) as-synthesized IBN-4 nanoparticles, (**b**) IBN-4 nanoparticles after surfactant removal (IBN-4-extracted), (**c**) IBN-4 nanoparticles modified with APTS groups (IBN-4-NH_2_), (**d**) IBN-4-NH_2_ nanoparticles modified with glutaraldehyde groups (IBN-4-NH-GA) and (**e**) IBN-4-NH-GA nanoparticles immobilized with HRP (IBN-4-HRP).

Fourier transform infrared spectroscopy (FT-IR) was performed to characterize the chemical modifications of the IBN-4 samples and their post-synthetic conjugations. The spectra of the as-synthesized and surfactant extracted IBN-4 samples are displayed in Figure 4. The bands at 1080 and 460 cm^−1^ confirms the Si-O-Si framework in all of the samples. Additionally, the typical bands observed at approximately 2900–3200 cm^−1^ were attributed to the asymmetric stretching vibrations of C-H in the aliphatic moieties of the surfactant. These bands vanished after oxidative extraction (Figure 4b), in contrast to those observed in Figure 4a, which confirmed that the surfactant was completely removed. However, the peaks of the Si–O–Si framework remained, which confirmed that the framework was not destroyed under oxidative reaction conditions. The C–H stretching bands at 2973 cm^−1^ and 2933 cm^−1^ appeared in the spectra of the amine functionalized IBN-4 samples (Figure 4c). The glutaraldehyde-conjugated IBN-4 displayed C=O (an aldehyde) stretching modes at or near 1667 cm^−1^ (Figure 4d). The spectrum of the HRP-loaded nanocarrier resulted in amide bands at 1452 and 1550 cm^−1^. In addition, the band at 1550 cm^−1^ represented the NH bending vibrations in the plane; the intensity of the peak at 1452 was much lower than before [72]. Thus, the FT-IR spectra indicated the sequential modifications of IBN-4 were successful.

### 2.1. Detection of Primary Amine Groups

**Figure 5:**
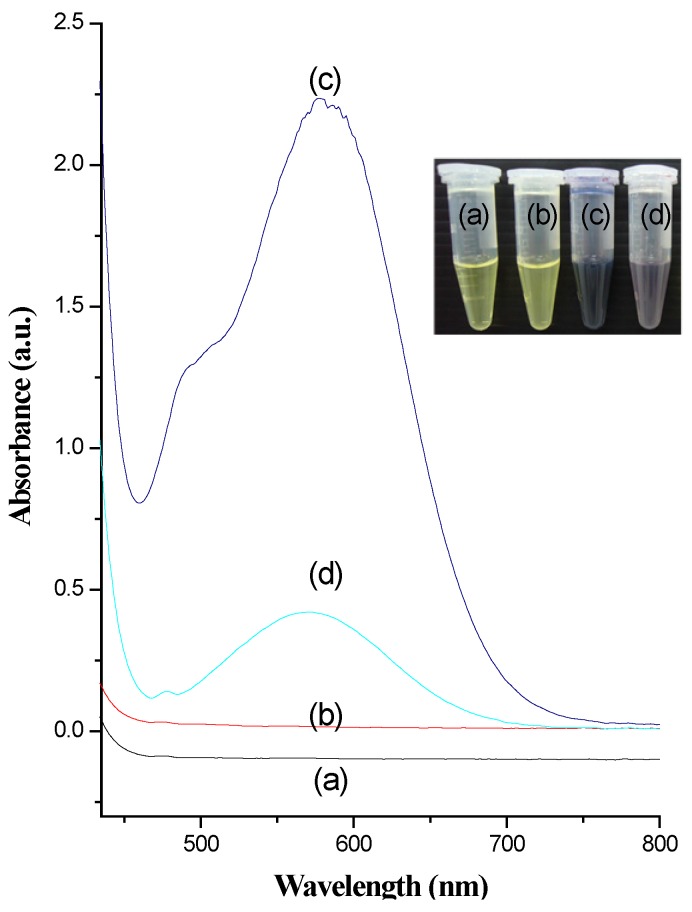
Ultraviolet–visible (UV-Vis) spectra and white-light sample images (inset) of (**a**) ninhydrin alone, (**b**) IBN-4 nanoparticles after surfactant removal, (**c**) IBN-4 nanoparticles modified with APTS groups (IBN-4-NH_2_) and (**d**) IBN-4 nanoparticles modified with glutaraldehyde groups (IBN-4-NH-GA).

The amino groups on the surface functionalized IBN-4 samples were quantitatively analyzed using UV-Vis spectrometry after treating the samples with ninhydrin to yield Ruhemann’s purple coloration that displayed an absorption band at 580 nm upon reaction with primary amines. Unmodified IBN-4 nanoparticles (Figure 5b) did not show any prominent peaks at 580 nm due to the absence of amine groups. However, IBN-4-NH_2_ samples exhibited a characteristic band at 580 nm (Figure 5c) along with the production of Ruhemann’s purple, as shown in the respective inset sample images. This phenomenon indicated that large amounts of amino groups were conjugated to the surfaces of the IBN-4 samples. Glutaraldehyde-conjugated IBN-4-NH_2_ nanomaterials (Figure 5d) showed very low absorption values for the active amine groups as confirmed by the lower absorption peaks at 580 nm; this observation could be explained by the reaction of the two terminal amino groups with glutaraldehyde moieties and their subsequent transformations into secondary amines.

### 2.2. Properties of Immobilized HRP

The specific activities per unit weight of enzymes for the HRP-immobilized nanoparticles were solely dependent on the method employed to immobilize the HRP molecules. We loaded HRP molecules using a typical two-step method (IBN-4-HRP) and utilized a one-pot synthetic approach (IBN-4-HRP (one pot)) to compare the resulting enzyme activities. The efficacies of delivery and the anti-tumor activities of the two methods were successfully compared. It was evident that enzyme immobilization onto IBN-4 nanomaterials using a two-step process (IBN-4-HRP (4144 units/g)) resulted in significantly higher yields than when the one pot approach was used (IBN-4-HRP (one pot)) (953.60 units/g)). The low enzymatic activities of the nanoparticles immobilized using the one-pot approach were attributed to the reaction between the amino groups of HRP and glutaraldehyde molecules, which formed undesirable enzyme complexes; these complexes may have caused the active sites to denature through saturation of lysine residues. These undesirable enzyme-bound complexes could be removed by washing steps, which limited enzyme loading in the functionalized IBN-4 nanoparticles. Consequently, this led to lower loading amounts with the one-pot synthetic approach (IBN-4-HRP (one pot *i.e.*, 9.87%) compared to IBN-4-HRP (two-step process *i.e.*, 13.13%)), as observed by UV-Vis spectroscopy with a Bio-Rad enzyme quantification assay. The long-term stability (stored at 4 °C) and the activity of HRP immobilized IBN-4 nanoparticles were also evaluated. Interestingly, over 90% of the original peroxidase activity of HRP was retained after two months. These results revealed that the stability of the HRP entrapped in IBN-4 increased without losing biological activity.

### 2.3. IAA-Dependent Cytotoxicity of HRP Encapsulated IBN-4

**Figure 6:**
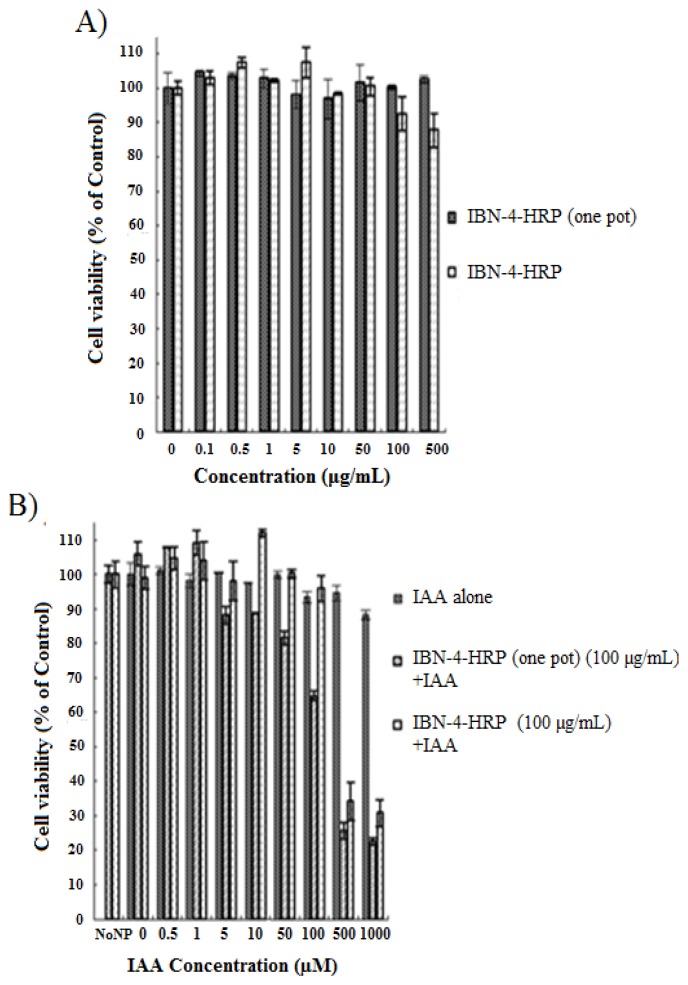

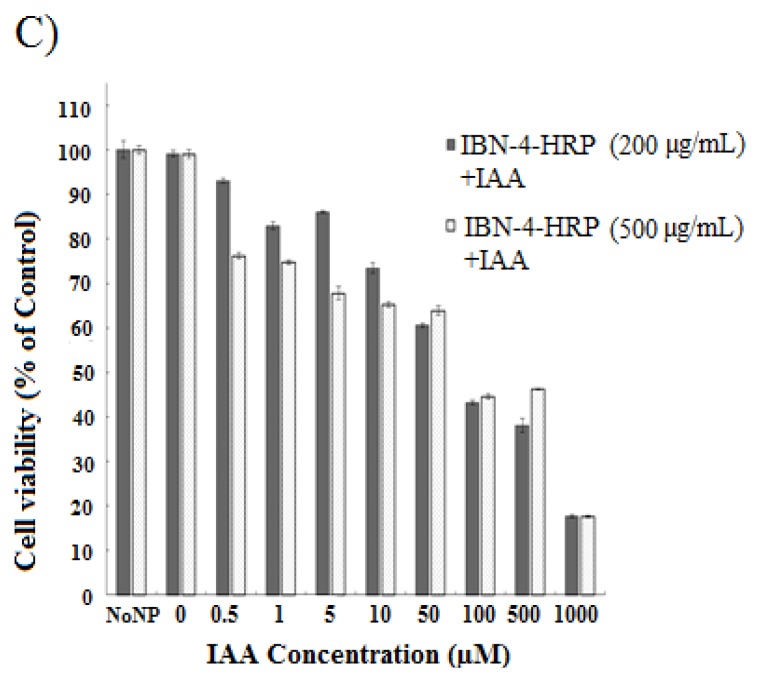
The viability of Human colon carcinoma (HT-29) cells: (**A**) treatment with IBN-4 loaded with Horseradish peroxidase (HRP) prepared via two synthetic routes; (**B**) treatment of IBN-4-HRP synthesized using two different routes in the presence or absence of indole-3-acetic acid (IAA) (IAA expressed in µM); and (**C**) treatment of various concentrations (200 and 500 μg/mL) of IBN-4-HRP synthesized in the presence or absence of IAA (IAA expressed in µM).

**Figure 7:**
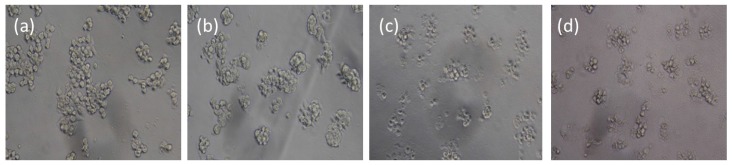
Bright field images of HT-29 cells without or with treatment of HRP-loaded IBN-4 nanoparticles and IAA: (**a**) Control (cells without treatment), (**b**) treatment with IAA (500 μM), (**c**) treatment with IAA (500 μM) and IBN-4-HRP (one-pot) (100 μg/mL) and (**d**) treatment with IAA (500 μM) and IBN-4-HRP (100 μg/mL).

The cytotoxic effects of using HRP-loaded IBN-4 in EPT were explored using an MTT (3-(4,5-dimethylthiazol-2-yl)-2,5-diphenyltetrazolium bromide) assay with a HT-29 (Human colon carcinoma) cell line (Figure 6), and the morphological changes were simultaneously observed using bright field microscopy (Figure 7). Previously, it was reported that the uptake of silica nanoparticles was fairly rapid and was observed after 2–3 h, with toxicity generally observed within 24 h [73]. To exclude the possible influence of enzyme-loaded nanoparticles on cell viability, various concentrations of HRP-loaded IBN-4 materials prepared using two synthetic routes were incubated for 24 h and the cell viability was evaluated. The tested concentration range was as high as 500 μg/mL, and the enzyme-loaded IBN-4 materials had no ostensible adverse effects on cell viability (Figure 6A). This demonstrated that the enzyme-loaded nanoparticles had no substantial cytotoxicity. Furthermore, the incubation of HT-29 cells with IAA alone did not show any signs of toxicity and, surprisingly, 90% of the cells were viable even at a concentration of 1000 μM (Figure 6B).

However, when IAA was combined with enzyme-loaded IBN-4, it became toxic to HT-29 cells (Figure 6B). Cell growth inhibition was observed to be 35% in IBN-4-HRP (one pot) (100 μg/mL) at 100 μM, whereas 70% of cell death was observed when the concentration of IAA was increased to 500 μM. A very similar dose-dependent cytotoxic pattern was observed in cells treated with IBN-4-HRP in combination with IAA. Nevertheless, the activity of the enzymes immobilized with the one-pot approach showed significantly higher activity than the particles immobilized with the typical two-step approach at lower concentrations; at higher concentrations, both samples exhibited nearly identical cytotoxic effects. This contrast in enzyme activity and cytotoxicity with respect to different synthetic approaches may have been due to the enzyme release characteristics during the prodrug activation process. Similarly, when treatment was continued at higher concentrations of IBN-4-HRP (200 and 500 μg/mL) (Figure 6C) in the presence of IAA (0–1000 μM), increases in the nanoparticle–enzyme complex concentration did not result in further changes in activity. This demonstrated that the enzyme prodrug therapy exclusively depends on the concentration of the prodrug and that the respective concentration (from 100 to 500 μg/mL) is suitable for activation of the prodrug.

The morphological changes of the HT-29 cells incubated with enzyme-loaded IBN-4 nanoparticles and IAA were also studied using bright field microscopy (Figure 7). HT-29 cells treated with enzyme-loaded IBN-4 nanomaterials (100 μg/mL) and IAA (500 μM) exhibited nuclear condensation and substantially shrunken morphologies (Figure 7c,d), suggesting the induction of apoptosis. No morphological changes were observed in cells treated with either IAA alone (Figure 7b) or with the untreated control (Figure 7a). These results were in accordance with observations from the cytotoxicity study and indicated that the enzyme-loaded IBN-4 materials have the potential to replace free-HRP in enzyme-prodrug cancer therapy.

**Figure 8:**
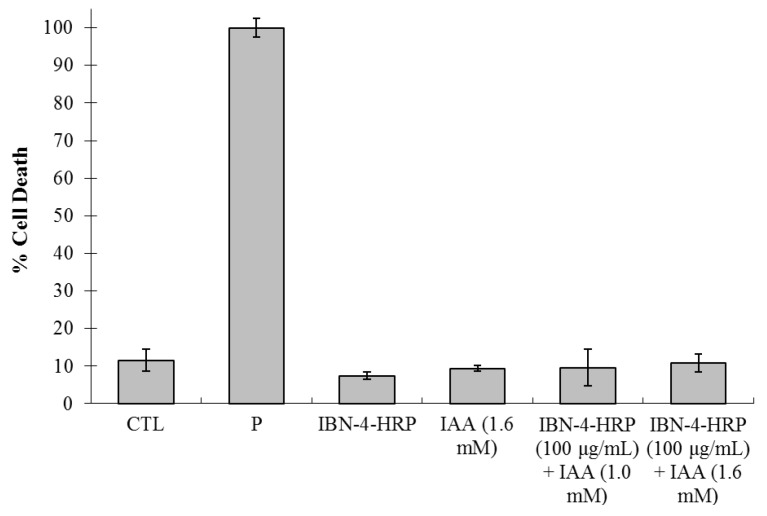
Cytotoxic estimates of IBN-4 nanocomposites using an lactate dehydrogenase (LDH) leakage assay. Control experiment (CTL), P represents positive control (treated with Triton x-100) with various treatments using combinations of IAA and IBN-4-HRP.

**Figure 9:**
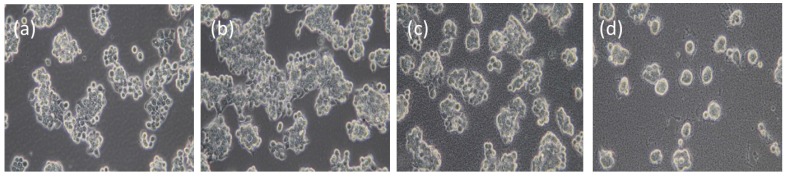
Bright field images of HT-29 cells with or without treatment of IBN-4-HRP and IAA: (**a**) HT-29 cells without treatment; (**b**) treatment with IAA (1.6 mM); (**c**) treatment with IBN-4-HRP alone; and (**d**) treatment with IBN-4-HRP and IAA (1.6 mM).

We used a lactate dehydrogenase (LDH) assay to measure the activity of the LDH enzyme that was released through cell membrane damage. For this experiment, maximum release of the positive control was obtained by treating the cells with 0.5% triton X-100 (Figure 8). Compared with the control experiment (CTL), treatment with IAA, (1.6 mM) enzyme-loaded IBN-4 materials, or a combination of the two did not result in a profound increase in the release of LDH even at doses as high as 100 μg/mL. From the results obtained, it was concluded that enzyme-loaded IBN-4 generated with either of the routes was highly biocompatible and retained membrane integrity by decreasing the amount of unexpected necrotic cell death. The majority of the anti-cancer drugs induce cell death through apoptotic or necrotic pathways. Induction of cell death by the apoptotic pathway confers many advantages over the necrotic pathway because the leakage of enzymes that results from the necrosis pathway usually causes an inflammatory response, which results in poor prognosis in cancer therapy. Furthermore, we analyzed the morphological changes of the HT-29 cells incubated with HRP-loaded IBN-4 and IAA by bright field microscopy (Figure 9). After treating the cells with IBN-4-HRP and IAA (1.6 mM), the morphologies of the cells became round and shrunk, which is a typical phenomenon observed for cell apoptosis. In comparison with the groups treated with IAA or IBN-4-HRP alone, the cell morphologies rarely had round shapes or shrunk, which demonstrates the low cytotoxicities of the individual treatment methods.

**Figure 10:**
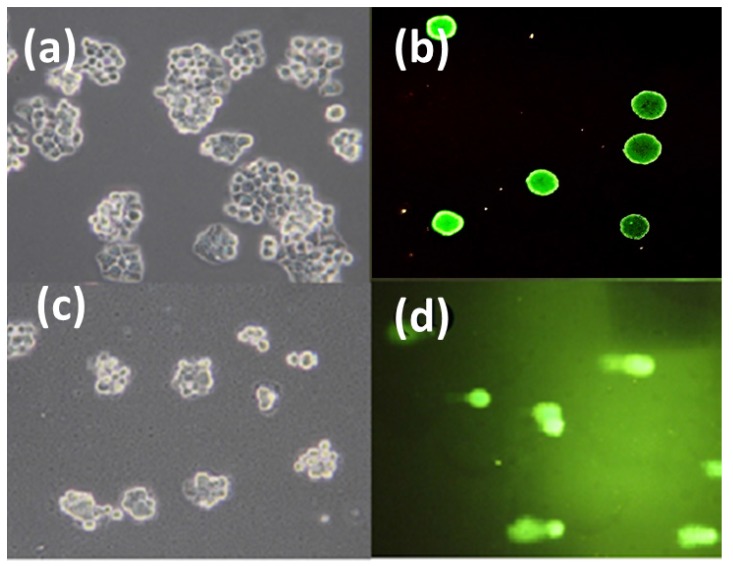
Microphotographs of bright field and fluorescent microscopic views of HT-29 cells: (**a**) Bright field view of untreated control cells (undamaged cells); (**b**) fluorescent microscopic view of untreated control cells (undamaged cells); (**c**) bright field view of cells treated with IAA (1 mM) and IBN-4-HRP (100 μg/mL) (shrunken morphologies); and (**d**) fluorescent microscopic view of cells IAA (1 mM) and IBN-4-HRP (100 μg/mL) (damaged cells with elongated tails).

Previous reports have suggested that the combination of IAA and HRP can induce apoptosis by activating p38 mitogen-activated protein (MAP) kinase and c-Jun N-terminal kinase (JNK). Furthermore, this combination was shown to activate caspase-8 and caspase-9 and to induce DNA damage [74]. To elucidate the mechanism of cell death, the DNA damage was investigated using a comet assay. Comet assays have been considered to be a very good technique for determining DNA damage and has been shown to have many advantages over existing methods that measure DNA fragmentation. The results of the comet assay showed significant DNA damage, which was indicated by the tail moment (Figure 10d) after treatment with enzyme loaded-IBN-4 materials (IBN-4-HRP) in the presence of IAA. Furthermore, the bright field images revealed profoundly shrunken cell morphologies when enzyme-loaded nanomaterials (IBN-4-HRP) were incubated along with IAA (500 μM) (Figure 10c), suggesting the induction of apoptosis. No morphological changes were observed in the control group (Figure 10a).

## 3. Experimental Section

### 3.1. Materials

Pluronic P123, tetraethyl orthosilicate (TEOS), ammonium perchlorate (AP), (3-aminopropyl)trimethoxysilane (APTS), 3-(4,5-dimethylthiazol-2-yl)-2,5-diphenyltetrazolium bromide (MTT), potassium bromide (KBr) (FT-IR grade), glutaraldehyde (GA), horseradish peroxidase (HRP) and 3-indole acetic acid (IAA) were purchased from Sigma-Aldrich^®^ (St. Louis, MO, USA). All other reagents and chemicals were analytical grade materials.

### 3.2. Characterization and Instruments

TEM images were captured on a Hitachi H-7100 (Hitachi High Technologies Corporation, Tokyo, Japan) instrument operating at 100 kV. Samples were prepared by dispersing them on carbon-coated copper (Cu) grids and drying them at room temperature. Fluorescence images were captured on an Olympus microscope hybridized with cooled color CCD of an Olympus DP73 (Olympus Corporation, Center Valley, PA, USA). The centrifuge used for nanomaterial syntheses and cell culturing processes at appropriate temperatures and rpm was an Hermle Z 36 HK (HERMLE Labortechnik GmbH, Wehingen, Germany) and a swing rotor Kubota KN-70 (Kubota Corporation, Tokyo, Japan), respectively. Thermogravimetric analyses were performed using a TGA Q50 V20, 13 Build 39 (Universal V4.5A TA Instruments, Lukens drive, New Castle, PA, USA). Dynamic light scattering (DLS) measurements to determine particle sizes and zeta potentials were performed using a Nano-HT Zetasizer 90 (Malvern Instruments Ltd., Worcestershire, UK). Ultraviolet-visible (UV-Vis) spectra were recorded on a Genequant-1300 series spectrophotometer (GE Healthcare Biosciences, Pittsburgh, PA, USA). MTT absorbance was recorded using an EnSpire Multi-label Plate Reader (Perkin Elmer, Santa Clara, CA, USA).

### 3.3. Synthesis of IBN-4 Type Mesoporous Silica Nanoparticles

IBN-4 type mesoporous silica nanoparticles were prepared by following a previously published method [75], where 1 g of pluronic acid P123 and 2.8 g of FC-4 (C_3_F_7_O(CFCF_3_CF_2_O)_2_CFCF_3_CONH(CH_2_)_3_N^+^(C_2_H_5_)_2_CH_3_I^−^) were dissolved in 160 mL of 0.02 M HCl at 45 °C. After complete dissolution of the surfactant, TEOS (4 g) was added and the reaction mixture was stirred at room temperature for 20 h. The pristine IBN-4 particles were collected by centrifugation at 12,000 rpm for 17 min, and the copolymer templates were further removed by ammonium perchlorate (AP) [76]. To create oxidative conditions, approximately 0.2 g of the IBN-4 solids was treated with 0.4 g of AP in 20 mL of a 10 M HNO_3_ solution under 100 °C for 12 h. The solid products were washed with dd-H_2_O and collected by centrifugation at 12,000 rpm for 17 min.

### 3.4. Synthesis of Amino-Functionalized IBN-4

Post-synthetic modification was carried out by adding 0.2 g of the IBN-4 solids in 30 mL of toluene and stirring the reaction mixture. A 0.3 mL aliquot of a pure APTS solution was added to the mixture and allowed to stir at 100 °C for 20 h. The amino-functionalized particles were collected by centrifugation at 12,000 rpm for 17 min and were washed twice using acetone and ethanol.

### 3.5. Detection of Primary Amines with a Ninhydrin Test

The procedure employed methods following a previously published report on the detection of amine-functionalized silica material [77]. Typically, 50 mg of the samples was mixed with 5 mL of ethanolic ninhydrin solution (0.2 M). The solution was stirred at room temperature for 25 min. Subsequently, the supernatant was collected to and centrifuged for 10 min. The absorbance of the supernatant was monitored at 580 nm by UV-Vis spectrometry.

### 3.6. Covalent Immobilization of HRP onto IBN-4 Materials (IBN-4-HRP)

Enzymes were immobilized onto IBN-4 nanomaterials following a previously published method [78]. Initially, 0.15 g of amine-functionalized particles was dispersed in 22.5 mL of a 0.1 M Na_2_HPO_4_-NaH_2_PO_4_ buffer solution (pH 8.0) through ultra-sonication. A 50% glutaraldehyde (GA) solution was added to the particles dispersed in buffer to reach a final volume of 0.1% (*v*/*v*). The reaction mixture was continuously stirred for one hour at room temperature and the GA-modified particles were collected by centrifugation. Later, the HRP buffer solution was prepared by dissolving 22.5 mg of HRP in 15 mL of a 10 mM Na_2_HPO_4_-NaH_2_PO_4_ buffer solution (pH 8.0). Finally, 0.15 g of the GA-modified IBN-4 particles was redispersed in the HRP buffer solution by ultra-sonication. The reaction mixture was then stirred at 10 °C for 2 h and collected by centrifugation. The unbound enzymes were removed by washing them once with a 10 mM Na_2_HPO_4_-NaH_2_PO_4_ buffer solution. The enzyme-bound particles were redispersed in 15 mL of a 10 mM Na_2_HPO_4_-NaH_2_PO_4_ buffer solution (pH-8.0) and stored at 4 °C.

### 3.7. Covalent Immobilization of HRP into IBN-4 Materials by One pot Process (IBN-4-HRP (One Pot))

The direct method of HRP immobilization onto IBN-4 nanomaterials followed a previously published method [79]. At first, 10 mg of amino-functionalized particles was dispersed in 5 mL of a phosphate buffer saline (PBS) solution (pH 7.4) by ultrasonic dispersion and was followed by the addition of 5 mL of a 2.5% glutaraldehyde solution. The reaction mixture was stirred for 2 h at room temperature and then 5 mg of HRP was added. The reaction mixture was allowed to stir at 4 °C for 24 h and the particles were recovered by centrifugation. The unbound enzymes were removed by washing them with PBS. Finally, the enzyme-conjugated particles were redispersed in 1 mL of pH 7.4 PBS and stored at 4 °C.

### 3.8. HRP Enzymatic Assay

The peroxidase activity of HRP was measured colorimetrically. The principle behind this measurement involves the peroxidation of pyrogallol to purpurogallin in the presence of HRP. A reaction mixture (3.00 mL) was prepared to contain the following final concentrations: 100 mM potassium phosphate buffer (pH-6.0), 0.5% (*w*/*w*) hydrogen peroxide, 5.0% (*w*/*v*) pyrogallol, and IBN-4-HRP (1 mg/mL). The reaction was allowed to continue for 5 min while the contents were mixed properly. The absorbance was measured at 420 nm with a UV-Vis spectrophotometer through calibration for 10 min at 30 °C. The absorbance of a blank sample was obtained using water. One unit/g of the IBN-4-HRP formed 1.0 mg of purpurogallin from pyrogallol at specific pH and temperatures. All the activity measurements were performed in triplicate.

### 3.9. Cytotoxicity Studies

Cell Culture: a HT-29 (colon carcinoma) cell line was cultured in Roswell Park Memorial Institute (RPMI)-1640 medium supplemented with 10% (*v*/*v*) fetal bovine serum (FBS). Cultures were maintained in a humidified incubator at 37 °C with 5% CO_2_ (carbon dioxide). Cell viability studies of the prepared particles were evaluated *in vitro* using a standard MTT (3-(4,5-dimethylthiazol-2-yl)-2,5-diphenyltetrazolium bromide) colorimetric assay. HT-29 cells were seeded in 96-well plates at a density of 1 × 10^4^ cells per well. After letting the cells adhere overnight, the cells were serum-starved in RPMI-1640 medium. The cells were incubated for 24 h and then treated with various concentrations (0.5–1000 µM) of 3-indoleacetic acid and enzyme-conjugated nanoparticles (0.1 mg/mL) and were incubated for another 24 h. Subsequently, 50 µL of an MTT solution (1 mg of MTT in 1 mL of PBS) was added and incubation was continued for another 4 h. The relative percentages of metabolically active cells relative to the untreated control cells were then determined based on the mitochondrial conversion of MTT to formazan. Finally, the medium was pipetted out and 150 μL of DMSO was added to wells to dissolve the crystals; then, the absorbances of the individual wells were measured with a Microplate Reader at 570 nm.

### 3.10. LDH Release Assay

HT-29 cells (1 × 10^5^ cells/well) were seeded in a 12-well plate. The cells were grown in 10% FBS-supplemented RPMI-1640. The cultured cells were treated with various sample combinations using IAA and IBN-4-HRP and were incubated for another 24 h. The levels of cytosolic LDH (lactate dehydrogenase) leakage were assessed to measure the extent of cellular membrane damage using a Sigma Tox-7 Kit (Sigma-Aldrich^®^, St Louis, MO, USA) following the manufacturer’s instructions. This kit spectrophotometrically determines LDH activity by measuring the intensity of the reduced formazan at 490 nm, which is directly proportional to the LDH activity. These measurements were performed with an ELISA reader. The results presented represent mean values from triplicate measurements. The results are given as fractions of LDH release compared to the positive controls, which consisted of 0.5% Triton X-100 (yielding 100% LDH release).

### 3.11. Comet Assay

The DNA comet assay was performed using a Trevigen’s Comet Assay^®^ kit (Trevigen, Inc. Gaithersburg, MD, USA) according to the manufacturer’s instructions. HT-29 cells were seeded at a density of 1 × 10^5^ cells/well in a 6-well plate and various treatments were performed. The plates were placed in an incubator for 20 h. After harvesting, the cells were pooled in a 1% low melting point agarose gel at a ratio of 1:10 (*v*/*v*). The gel was run in accordance with the manufacturers’ instructions for 20 min at 21 volts and 350 mA. The gel was then washed with water and ethanol to reanneal the DNA; eventually, the smear was stained with SYBR Green. DNA comets were considered to be markers of genotoxic effects. The images were captured using Olympus fluorescence image analyses.

## 4. Conclusions

To improve the enzymatic activity and stability of nanoparticles for use in therapeutic applications, the fragile enzyme molecules required immobilization on solid supports to prevent degradation and unfolding of the proteins. In this study, we immobilized an HRP enzyme onto nanochannels of mesoporous silica nanoparticles to demonstrate their ability to activate an anticancer prodrug (indole-3-acetic acid). The immobilization of HRP and activity experiments indicated that the confined spaces in mesoporous silica could maintain HRP microenvironments and therapeutic activities. The large pores of the mesoporous silica (IBN-4) nanocontainers were suitable for immobilizing HRP enzymes through covalent bonds. The conditions employed for enzyme loading using a typical approach via two chemical steps generated more favorable critical parameters for enzyme orientation and specific activities than the one-pot approach. It is expected that IBN-4 materials with large surface areas, pore sizes, and high biocompatibility can be used to enhance the stability of HRP enzymes and increase the diffusion rates of catalytic substrates, which can be used in EPT for cancer therapeutic applications.
